# Effects of *In Vitro* Zinc Sulphate Additive to The Semen Extender on Water Buffalo (*Bubalusbubalis*) Spermatozoa before and after Freezing 

**Published:** 2014-11-01

**Authors:** Kamran Dorostkar, Sayed Mortaza Alavi Shoushtari, Amir Khaki

**Affiliations:** 1 Department of Clinical Sciences, Faculty of Veterinary Medicine, Azad University, Sanandaj Branch, Sanandaj, Iran; 2 Division of Theriogenology, Department of Clinical Sciences, Faculty of Veterinary Medicine, Urmia University, Urmia, Iran

**Keywords:** Semen, Zinc, Buffalo

## Abstract

**Background:**

The objective of the study was to investigate the effects of *in vitro* zinc sulphate additive to semen extender on sperm parameters (progressive motility, viability, membrane integrity and DNA stability) after cryopreservation.

**Materials and Methods:**

In this Prospective longitudinal laboratory study, semen samples of 5 buffalo bulls of 3-5 years old were collected at 5 different occasions from Iran, Urmia during summer and autumn 2011, 25 samples were used in each treatment. Sperm progressive motility, viability and abnormal morphology were measured before and at 0.5 (T_0_), 1(T_1_) and 2(T_2_) hours after diluting semen(1:10 v/v) in Tris-citric acid based extender (without egg yolk and glycerol) at 37˚C containing none (control group), 0.072, 0.144, 0.288, 0.576 and 1.152 mg/L zinc sulphate to investigate dose and time effects. Next, a Tris-citric acid-egg yolk-glycerol extender (20% egg yolk and 7% glycerol) containing the same amount of zinc sulphate was prepared, diluted semen (1:10 v/v) was cooled and kept into a refrigerated chamber (4˚C) for 4 hours to equilibrate. Sperm progressive motility, viability, abnormal morphology, membrane integrity and DNA damage were estimated.The equilibrated semen was loaded in 0.5 ml French straws and frozen in liquid nitrogen. Later, the frozen semen was thawed and the same parameters as well as total antioxidant capacity (TAC) of the frozen-thawed semen were determined.

**Results:**

The results showed that zinc sulphate additive at the rate of 0.288 mg/L gave a higher protection of sperm progressive motility (53.7 ± 1.8% vs. 40.5 ± 1.7%), viability (70.8 ± 1.8% vs. 60.1 ± 1.5%), membrane integrity (67.3 ± 1.6% vs. 56.6 ± 1.7%), DNA stability (10.1 ± 0.47% vs. 11.8 ± 0.33% damaged DNA) through the process of dilution, equilibration and freeze-thawing and caused a higher TAC level (81 ± 3.3% vs. 63 ± 3.2 µmol/L) after freez-thawing compared to the control group. Adding 0.576 and 1.152 mg/L zinc sulphate, however, was deleterious to the sperm and significantly reduced the studied sperm parameters.

**Conclusion:**

Adding 0.288 mg/L zinc sulphate to the extender, compared to the control group, gives a better sperm preservation upon freezing processes which in turn, may results in higher semen fertility. But, addition of higher zinc sulphate concentrations (0.576 and 1.152 mg/L) are detrimental to buffalo spermatozoa.

## Introduction

Semen cryopreservation is an important section of artificial insemination programs (1), it allows preservation of semen fertility for a long time. In this procedure, however, many spermatozoa lose their motility and other parameters which lead to a low fertility. A number of studies have demonstrated membrane lipid peroxidation as one of the causes of defective sperm function in liquid semen preserved at 4˚C (2) and in cryopreserved semen (3, 4). Some attempts (5-7) have been made to preserve sperm parameters, particularly sperm motility, by adding some elements (zinc, copper and selenium) (8) and materials (vitamins, antioxidants, amino acids and coenzymes) to the semen before freezing.

Zinc is one of the important trace elements in the body, while its deficiency causes infertility in most animals due to disorders of testes development, spermatogenesis (9), steroidogenesis through gonadotropic hormones secretion (10), genetic expression of steroid receptors (11), testosterone synthesis and function through Zn-dependent metalloenzyme 5α-reductase (12) and serum cholesterol level adjustment (13). The total zinc content in semen from mammalians is high and zinc has been found to be critical to spermatogenesis (14, 15) and sperm concentration (16).

Zinc ions affect the expression of some germ-cell specific-genes during the course of spermatogenesis in sheep (17). Zinc supplementation leads to improved fertility in zinc deficient animals by increasing concentration and motility of spermatozoa (18) and sperm membrane integrity and reducing sperm DNA damage in human subjects (19). Zinc also contributes to the stability of sperm chromatin and repair of DNA damage (20). Mechanisms responsible for DNA strand break and changes in the histone to protamine ratio in DNA of zinc deficient men has been described in detail (20). Zinc influences the fluidity of lipids, and thus the stability of biological membranes. It is involved in the formation of free oxygen radicals and may play a regulatory role in the process of capacitation and the acrosome reaction (21), sperm nuclear chromatin condensation and acrosinactivity (22). Zinc, at high concentrations in particular, may depress oxygen uptake (cell respiration) in the sperm cell and influence the sperm motility (23), zinc antioxidant capacity has been reported by many authors (24, 25). Alavi-Shoushtari et al. observed that buffalo semen samples with higher seminal plasma zinc content (161.07 ± 1.36 vs. 136.42 ± 4.96 mg/L) had higher sperm motility, viability as well as lower abnormal morphology (26) and total antioxidant capacity (1.57 ± 0.01 vs. 1.23 ± 0.05 mmol/L) (27).

Information on the *in vitro* effects of zinc sulphate on buffalo bull spermatozoa is scarce. This prospective longitudinal laboratory study was conducted to investigate the effects of *in vitro* zinc supplementation on the progressive motility, viability, sperm membrane integrity and DNA stability of the spermatozoa in buffalo bulls’ semen before and after semen equilibration and freezing with the aim of finding a practical protocol to have an improved semen quality after freeze-thawing.

## Materials and Methods

### Semen collection and processing

In this prospective longitudinal laboratory study, semen samples of 5 buffalo bulls of 3-5 years old kept in Buffalo Breeding Center of North-West of Iran, Urmia (37° 33' N, 45° 4' E), were collected by artificial vagina at 5 different occasions at weekly intervals during the late summer and autumn 2011. A total number of 25 samples were used in each examination. Semen samples were immediately transferred into a 37℃ incubated at 37˚C for 40 minutes. Aftl (appearance, homogeneity, density, color and volume) examination. Sperm gross and progressive motility, viability, and abnormal morphology of semen were evaluateded. Thin, watery specimes with low quality were discarded. These parameters (except gross motility) were measured within 0.5(T_0_), 1(T_1_) and 2(T_2_) hours after diluting semen (1:10 v/v) in the Tris-citric acid extender traditioallyused in the center [(without egg yolk and glycerol), pH=7.1, osmotic pressure≡300 mosmolkg^-1^ and all the chemicals were purchased from Merck Co., Germany]. The extender consisting of Tris 2.66 g, glucose 1.2 g, citric acid 1.39 g, cysteine 0.139 g and distilled water in total volume of 100 ml that contained none (control, without zinc sulphate), 0.072, 0.144, 0.288, 0.576 and 1.152 mg/L zinc sulphate (ZnSO_4_, 7H_2_O, ScharlauChemie S.A., Sentimental, Spain). Next, a Tris-citric acid-egg yolk-glycerol extender (above mentioned extender with 20% egg yolk and 7% glycerol, added in one step, as protectant and without antimicrobial agents) containing the same amounts of zinc sulphate was prepared at room temperature. The semen samples were diluted at a rate of 1:10 v/v, (an approximate count of 12×10^6^ sperm cells/mL), cooled to 4℃ within 2 hours, transferred to the equilibrium chamber of 4℃ and left for 4 extra hours to equilibrate. Sperm parameters (progressive motility, viability, membrane integrity and DNA damage) were estimated in equilibrated semen and semen was then loaded in 0.5 ml French straws and frozen over liquid nitrogen in static vapor up to .120℃ within 25 minutes before being plunged in the liquid nitrogen (28) and stored until further analysis. Later, the frozen semen was thawed in 37℃ water bath for 30 seconds, and the same parameters, as well as total antioxidant capacity (TAC) of the frozen-thawed semen, were determined.

### Sperm functional assays

#### Sperm progressive motility, viability and abnormal morphology

Sperm progressive motility was estimated using a computer assisted system of analysis (CASA) [Hoshmand Fannavar (HF) CASA, version 6, Amirkabir Medical Engineering Co, Tehran]. After the samples were stained using eosin-nigrosin staining method (29), sperm viability was estimated by counting live and dead spermatozoa using a light microscope (Olympus BX41, Japan), at least 200 sperm cells were examined on each slide. Sperm cell abnormal morphology was also estimated.

#### Plasma membrane integrity

Hypo-osmotic swelling test (HOST) was used to examine membrane integrity of spermatozoa before and after freezing according to a method of Jeyendran et al. (30) as described by El-Sisy et al. (31). In brief, the hypo-osmotic solution (osmotic pressure≡190 mosmol kg^-1^, Osmomat 030, Nr. 951211, Gonotec, Germany) was prepared by dissolving 0.73 g sodium citrate and 1.35 g fructose in 100 ml of distilled water. Hypo-osmotic solution (500 μl) was mixed with 50 μl of semen and incubated at 37˚C for 40 minutes. After incubation, a drop of semen sample was examined under a phase-contrast microscope (×400) (Olympus BX31, Olympus Optical Co., Japan) and 200 spermatozoa were counted in at least 5 different fields for their swelling characterized by coiled tail indicating intact plasma membrane, which could be differentiated from abnormally coiled tails previously estimated.

### Sperm DNA damage

DNA damage was detected using acridine orange staining technique, according to the method of Katayose et al. (32). Briefly, first, spermatozoa were smeared on the glass slide. After being air dried, the samples were treated with acid alcohol (methyl alcohol-glacial acetic acid 3:1, vol/vol) for 2 hours. Immediately after air drying, approximately 1 mL working solution including 0.019% acridine orange [3, 6-bis (dimethylamino) acridine and hemi (zinc chloride) salt (Sigma Chemical Co., St. Louis, MO, USA)] was mounted on each glass slide for 5 minutes at room temperature and the samples were then washed with distilled water. The samples were observed under a fluorescence microscope (Model GS7, Nikon Co., Japan) immediately after placing a coverslip on the slide. A total of 100 to 200 spermatozoa were observed and classified, like green (intact DNA) or red (damaged DNA), based on differences in their fluorescent color.

### Total antioxidant capacity (TAC)

TAC of the frozen-thawed semen was assayed in duplicates using a commercial kit (Antioxidant Capacity Assay Kit, Cayman Chemical Co., Ann Arbor, MI, USA).

### Statistical analysis

The data obtained from 25 semen samples from 5 bulls (total number of 125 assays for each estimation) were analyzed using SAS (Version 9.2, SAS Institute Inc., Cary NC.). Prior to analyses, data were checked using box plots to examine for errors and outliers. No outlier was detected. The parameters were analyzed using a linear mixed model (PROC MIXED) with the REPEATED command. The Kenward-Roger procedure was used to approximate the denominator degrees of freedom. The residuals were assessed visually by quantile-quantile plots for testing of the normality and also predicted values were plotted versus residuals to assess the homogeneity of variance. To meet the assumptions of the tests, the data for all parameters, except than that for TAC, were transformed by applying square root transformation. The initial model for each parameter included treatment, stage and their interactions as fixed effects and buffaloes nested in treatment were considered as random effect. When a non-significant interaction term was detected, the model was re-run with the interaction effect excluded from the model. For each parameter several covariance structure between repeated measures were examined and the model which provided better fit according to minimum Akaike’s Information Criterion (AIC) was used. Differences between least squares means were determined using the DIFF option and Bonferroni’s correction was applied to pair-wise comparisons. All reported values are least squares means and statistical mean and standard error of mean (SEM). Effects were declared significant at p<0.05. This study was approved by the Ethic Committee of Urmia University.

## Results

### Freshly diluted semen

The results are summarized in tables 1 and 2. Adding 0.288 mg/L zinc sulphate to the extender preserved the sperm motility at T_1_ and T_2_ compared to controls (85.4 ± 1.2% vs. 84.8 ± 1.5% at T_0_, 85.5 ± 1.1% vs. 80.8 ± 1.3% at T_1_ and 85.7 ± 1.1% vs. 77.4 ± 1.5% at T_2_) which was significant (p<0.05) at T_1_ and T_2_ indicating that the highest value belongs to only at T_2_, whereas shows a significant effect on sperm viability (86.3 ± 0.9%, p<0.05, Table 1). Immediately after adding 0.576 and 1.152 mg/L zinc sulphate (T_0_) to the extender, the sperm motility (from 84.8 ± 1.5% in control to 80.7 ± 1.7% and 75.2 ± 1.2 % respectively) and viability (from 86.1 ± 1.2% in control to 83.5 ± 1.6% and 77.8 ± 1.2% respectively) were significantly (p<0.05) decreased as compared with the controls and continued decreasing as time passed (p<0.001, Table 1). Sperm abnormal morphology did not show significant variation.

### Equilibrated diluted semen

In equilibrated diluted semen the highest sperm progressive motility (80.7 ± 1.9%), viability (84.1 ± 1.0%) and sperm membrane integrity (83.3 ± 0.9%) values were observed in 0.288 mg/L zinc sulphate, the number of intact DNA cells (green cells) in this diluted solution was not statistically different from control. Adding 1.152 mg/L zinc sulphate to the extender, however, significantly (p<0.001) reduced motility, viability and membrane integrity while increasing sperm cell DNA damagerate (Table 2).

**Table 1:** Effects of different zinc sulphate concentrations (mg/L) on the motility and viability (LS mean ± SEM) of spermatozoa in freshly diluted semen


Parameter	Time	Treatment						SE	P value		
		Control	0.072	0.144	0.288	0.576	1.152		TRT	STG	TRT×STG

**Motility**	T_0_	84.8± 1.5^a^^b^	84.2± 1.4^a^^b^	85.0± 1.8^a^^b^	85.4± 1.2^a^	80.7± 1.7^b^	75.2± 1.2^c^	0.0025	<0.0001	<0.0001	<0.0001
	T_1_	80.8± 1.3^a^^c^	81.0± 1.4^a^^c^	83.5± 1.6^a^^b^	85.5± 1.1^b^	78.2± 1.7^c^	71.7± 1.2^d^	0.0025			
	T_2_	77.4± 1.5^a^**	78.9± 1.2^b^^c^**	81.6± 1.4^b^	85.7± 1.1^c^	72.7± 1.0d***^+^	67.9± 1.2e***^+^	0.0025			
**Viability**	T_0_	86.1± 1.2^a^^b^	86.3± 1.2^a^^b^	87.3± 1.5^a^	87.2± 1.0^a^^b^	83.5± 1.6^b^	77.8± 1.2^c^	0.0016	<0.0001	<0.0001	0.0220
	T_1_	83.8± 1.5^a^^b^	84.2± 1.2^a^	85.6± 1.4^a^	86.8± 1.0^a^	80.2± 1.6^b^	74.0± 1.3^c^*	0.0016			
	T_2_	81.7± 1.1^a^^c^*	80.2± 1.4^a^^c^**^+^	83.5± 1.2^a^^b^	86.3± 0.9^b^	78.7± 1.2^c^*	71.7± 0.9^d^***	0.0016			


T_0_; 0, T_1_; 60, T_2_; 120 minutes after diluting semen, a, b, c, d, e ; Signify a significant difference (p<0.05) within the same row, *; Signifies a significant difference (p<0.05) with T_0_ values, **; Signifies a significant difference (p<0.01) with T_0_ values,***; Signifies a significant difference (p<0.001) with T_0_ values, +; Denotes a significant difference (p<0.05) with T_1_ values, LS; Least square, TRT; Treatment and STG; Stage.

**Table 2:** Effect of different zinc sulphate concentrations (mg/L) on sperm parameters (LS mean ± SEM) after equilibrium and thawing* time


Parameter	Stage	Treatment						SE	P value		
		Control	0.072	0.144	0.288	0.576	1.152		TRT	STG	TRT×STG

**Motility**	EQ	71.4± 1.9^a^	73.5± 1.8^a^^b^	78.3± 1.6^b^^c^	80.7± 1.9^c^	64.1± 1.6^d^	56.9± 1.3^e^	0.0081	<0.0001	<0.0001	<0.0001
**(%)**	TW	40.5± 1.7^a^	43.2± 2.0^a^^b^	47.8± 1.9^b^	53.7± 1.8^c^	32.9± 1.7^d^	27.6± 1.6^e^	0.0081			
**Viability**	EQ	78.0± 1.5^a^	79.1± 1.3^a^	80.4± 1.2^a^	84.1± 1.0^b^	77.4± 1.1^a^	73.5± 1.3^c^	0.0036	<0.0001	<0.0001	<0.0001
**(%)**	TW	60.1± 1.5^a^	62.0± 1.6^a^^b^	65.7± 1.8^b^	70.8± 1.8^c^	59.6± 1.8^a^	53.7± 1.9^d^	0.0036			
**Membrane**	EQ	79.0± 1.2^a^^c^	79.4± 1.3^a^^c^	81.7± 0.9^a^^b^	83.3± 0.9^b^	77.8± 1.1^c^	73.8± 1.5^d^	0.0036	<0.0001	<0.0001	<0.0001
**Integrity (%)**	TW	56.6± 1.7^a^	58.9± 1.7^a^	62.1± 1.8^b^	67.3± 1.6^c^	54.9± 1.7^a^	48.8± 2.2^d^	0.0036			
**Damaged**	EQ	3.0 ± 0.25^a^	3.4 ± 0.21^a^	3.4 ± 0.23^a^	3.0 ± 0.25^a^	3.5 ± 0.24^a^^b^	4.14± 0.21^b^	0.0025			
**DNA (%)**	TW	11.8 ± 0.33^a^	11.6 ± 0.43^a^	10.3± 0.49^b^	10.1± 0.47^b^	12.2± 0.33^a^^c^	13.3± 0.26^c^	0.0025	<0.0001	<0.0001	0.3084
**TAC (µmol/L)**	TW	63 ± 3.2^a^	64 ± 3.7^a^	77 ± 3.4^b^	81 ± 3.3^b^	57 ± 2.7^a^^c^	49 ± 2.4^c^				


*; 30 seconds in a 37˚C water bath, EQ; After equilibrium, TW; After thawing, TAC; Total antioxidant capacity, a, b, c, d, e; Signify a significant difference (p<0.05) within the same row, LS; Least square, TRT; Treatment and STG; Stage.

### Frozen-thawed semen

In frozen-thawed semen, the highest sperm motility (53.7 ± 1.8%, p=0.000), viability (70.8 ± 1.8 %, p=0.001) and sperm membrane integrity (67.3 ± 1.6%, p<0.05) values, were observed in 0.288 mg/L zinc sulphate, indicating that this concentration is significantly higher than the other treatments. Furthermore, the least number of DNA damaged cells (p<0.01) was obtained in this treatment (Table 2). Moreover, adding 0.144 and 0.288 mg/L zinc sulphate to the freezing extender, significantly (p<0.05) increased the total antioxidant capacity of the frozen-thawed semen as compared to the control (77 ± 3.4 and 81 ± 3.3 ìmol/L vs. 63 ± 3.2 ìmol/L, respectively).

## Discussion

Sperm motility and fertility is reduced by sperm processing and cryopreservation involved in the semen preservation in artificial insemination programs (33-35), while the antioxidant defense capacity of the semen is also decreased (36-38). Furthermore, antioxidants supplementation of semen extenders during liquid storage or cryopreservation of the bull (5) and the boar (39) spermatozoa and its beneficial effects have been reported. Therefore, this study was designed to investigate the effects of different doses of zinc sulphate supplementation added tothe extender on the quality of the sperm during freezing.

The extender used in this study was Tris-citric acid base extender that had been used in the Buffalo Breeding Center for some years but the egg yolk, glycerol and antibiotics were not added to it in the first stage, in order not to interfere with the examination and in the view that they might have some effect on the sperm parameters. Andrabi (40) in a review of several studies concluded that Tris–citric acid might provide the best satisfactory buffering system to improve the post-thaw freezability and motion characterictics of buffalo spermatozoa. Akhter et al. (41) compared effects of soya lecithin based extender (Bioxcell®), Tris-citric acid egg yolk, egg yolk-citrate and skim milk extenders on buffalo spermatozoa stored at 5˚C for 5 days and observed that motility, viability and plasma membrane integrity of buffalo bulls were similar in Tris-citric acid egg yolk, milk and Bioxcell on the first and third day of storage but on the fifth day they were better in Bioxcell. However, Rasul et al. (42) reported that Tris-citrate was better than Tris-citric acid, Tris-Tes and Tris-HEPES buffers in terms of improving post-thaw motion characteristics of buffalo spermatozoa.

In this study a CASA was used for the sperm progressive motility evaluation, but since the system was not calibrated for buffalo sperm, progressive motility of 25 semen samples were examined visually coincident with the system evaluation and the results were compared. The difference of two readings was less than 3%. So, only this parameter of motility was used in the study. The other parameters of motility were not used because their reliability for buffalo semen was not assured.

Sperm progressive motility, viability and abnormal morphology were evaluated immediately after the semen being diluted with the extender containing different dozes of zinc sulphate at different time intervals in order to assess the dose and time effect of zinc sulphate on spermatozoa quality. In all the examinations, all the treated samples were compared with their own control which had no zinc sulphate in its extender.

In selecting zinc sulphate dose two points were considered: 1. molecular weight (MW) of "zinc sulphate, 7H_2_O" is nearly 288 (287.57) grams with 65 gram zinc content, 2. Storage media, commercially prepared for *in vitro* fertilization (IVF) (Ham’s F-10), usually contains 0.0288 mg/L (1:10000 MW) zinc sulphate, 7H_2_O in their composition (43). The dose of 0.288 mg/L (1:1000 MW) zinc sulphate, 7H_2_O, 10 times more than that in storage media, was selected as a base to creat a zinc sulphate dose high enough to affect the spermatozoa;it was two times divided into halves [0.288:2(0.144) and 0.288:4(0.072) mg/L) or doubled 0.288×2(0.576) and 0.288×4 (1.152) mg/L] for dose selection in this study to gain a reasonable range of zinc sulphate concentrations in the extender. This selection worked well in the study since low doses (0.072 and 0.144 mg/L) were not as effective as 0.288 mg/L dose and high doses (0.576 and 1.152 mg/L) showed detrimental effects.

The present study revealed that supplementation of zinc sulphate improved the quality of freshly diluted and frozen semen of buffalo bulls as compared to the non-supplemented control group. An earlier study on buffalo semen by Alavi-Shoushtari et al. (26) showed significant correlations between zinc content of seminal plasma and sperm parameters including progressive motility and viability, suggesting that zinc ions may be positively correlated with semen quality.Some authors have suggested that zinc, as an antioxidant agent, particularly as a co-factor of copper/zinc superoxide dismutase (Cu/Zn SOD), plays a major role in the protection of spermatozoa against peroxidative damages of and reactive oxygen species (ROS) (44). This is shown by adding Zn to the testicular cell culture and increased enzymatic activity of Cu/Zn SOD (45). Our results revealed that adding zinc sulphate to semen extender improves semen TAC after freezing, in a dose dependant manner. These results were in consistent with the study of Omu et al. (19), who reported zinc therapy-associated improvement in sperm parameters includes an increase in the seminal antioxidant capacity and reduction of oxidative stress status. Alvarez and Storey (46) demonstrated that cryopreservation enhanced lipid peroxidation due to the loss of SOD activity during the freezing process. A better preserved sperm progressive motility, viability and membrane integrity after semen equilibration and freezing observed in this study could be attributed to increased antioxidant capacity of zinc ions, as reported by Hidiroglou et al. (47). Furthermore, this antioxidant activity might have been responsible for lower cases of DNA damage observed in this study. This could also be the effect of zinc antioxidant capacity on ROS released from the sperm mitochondria or on preventing production of lipid peroxidation products during freezing, which could cause DNA damage (DNA strand breakage) (20).

As most of sperm functions originated from the membrane, zinc may improve sperm functional capacity by creating a favorable environment (19, 48). In this study, the higher membrane integrity rate after adding 0.288 mg/L of the extender before and after semen freezing may be due to membrane stabilizing effect of zinc which is the result of interacting with some functional group of the intrinsic component of sperm membrane that has been reported by Sorensen et al. (14). We also observed a high percentage of membrane integrity, progressive motility and viability in test group of 0.288 mg/L, before and after freezing. As reported by Kumar et al. (49), sperm motility and viability are dependent upon membrane transport rate affected by zinc concentration.

A low percentage of motile sperms after adding high levels of zinc sulphate (0.576 and 1.152 mg/L) to the extender observed in this study could be due to an elevated free zinc fraction and its subsequent uptake by spermatozoa (50) and reduction of oxygen consumption, since high levels of zinc in semen impairs the oxygen consumption of sperms (14). This elevated free zinc fraction may be accounted for lower viability and membrane integrity rate and for higher cases of DNA damage as recorded in this study.

In this study zinc is believed to be important for membrane and chromatin stability and sperm motility (47). The high sperm viability values obtained by adding 0.144 and 0.288 mg/L of zinc sulphate may be due to the membrane stabilizing action of zinc to prevent leakage of enzymes, proteins and other vital components of the sperm in order to extend the functional life of sperm (49). Betteger and O’Dell (51) have reported that zinc stabilizes ribosomes, lysosomes, DNA and RNA that result in survival and normal functioning of the sperm. They further found that zinc protects sperm from free radicals that induce damages through their scavenging properties.

Storage time affected quality of the freshly diluted semen by deteriorating sperm progressive motility and viability which was more evident in the control group and low doses of zinc sulphatesupplementation groups, while adding 0.288 mg/ml zinc sulphate to the extender showed significantly better result. In equilibrated semen some spermatozoa did not tolerate the process. This was evident by lower semen quality (Table 2). Many sperms were damaged during the process of freezing which led to lower percentage of sperm progressive motility, viability, and membrane integrity, lower total antioxidant capacity and higher DNA damaged cells. Adding 0.288 mg/ml zinc sulphate to the extender significantly reduced the cell damage during freezing process, while the lower doses were not so effective and higher doses had adverse effect.

## Conclusion

The results showed that 0.288 mg/L zinc sulphate improve to the sperm quality (progressive motility, viability, membrane integrity and total antioxidant capacity) preservation upon freezing processes, however, we suggest a bigger sample population to achieve a definite statement. Our finding also revealed that zinc affects the cell membrane and leads to a lower degree of sperm DNA damage after semen freeze-thawing, which in turn, results in higher semen fertility. However, addition of higher zinc concentrations (0.576 and 1.152 mg/L) are detrimental to spermatozoa.
